# Association of frailty index with congestive heart failure, all-cause and cardiovascular mortality among individuals with type 2 diabetes: a study from National Health and Nutrition Examination Surveys (NHANES), 1999–2018

**DOI:** 10.1186/s13098-023-01165-z

**Published:** 2023-10-24

**Authors:** Yu-Nan Qin, Xiao-Pu Zheng

**Affiliations:** 1 Department of Cardiology, The First Affiliated Hospital of Xi’an Jiaotong University, 277 Yanta West Street, Xi’an, 710061 Shaanxi People’s Republic of China; 2 Key Laboratory of Molecular Cardiology of Shaanxi Province, Xi’an, Shaanxi China

**Keywords:** Frailty index, Congestive heart failure, Diabetes, Mortality

## Abstract

**Background:**

Both type 2 diabetes mellitus (T2DM) and frailty are strongly associated with congestive heart failure (CHF). Individuals with T2DM and CHF have a high frailty burden. The association of frailty with HF, all-cause, and cardiovascular mortality in patients with T2DM has not been thoroughly explored.

**Methods:**

This study included 2894 adults with T2DM from the National Health and Nutrition Examination Survey (NHANES) database over ten cycles (1999–2018) and followed up for all-cause and cardiovascular mortality through 31 December 2019. The frailty index (FI) was calculated using a 46-item deficit model to assess frailty status. Weighted multivariable logistic regression was performed to explore the relationship between frailty and CHF in patients with T2DM. Weighted restricted cubic splines were used to evaluate the non-linear relationship between FI and outcome. All-cause mortality and cardiovascular mortality association with FI was assessed using the Kaplan–Meier curve and COX proportional hazards regression accounting for sampling weights. Subgroup and sensitivity analyses were performed to evaluate the robustness of the results.

**Results:**

After the adjustment of essential confounders, a higher frailty index in T2DM was associated with increased odds of CHF (odds ratio [OR] for per 1-SD increase, 2.02, 95% confidence interval [CI] 1.67–2.45;* P* < 0.0001). The presence of frailty T2DM (OR, 3.60; 95% CI 2.34–5.54; *P* < 0.0001) was associated with a significant increase in the prevalence of CHF compared to non-frailty T2DM in a fully adjusted model. During the median follow-up of 6.75 years, per 1-SD increase in FI was associated with a 41% higher risk of all-cause mortality and a 30% higher risk of cardiovascular mortality after being adjusted for all confounders. Similar results were observed when sensitivity analyses were performed. There was also a non-linear relationship between FI and all-cause mortality. In a weighted multivariate COX proportional model adjusted for full confounders, frailty T2DM increased all-cause (HR, 1.86; 95% CI 1.55–2.24; *P* < 0.0001) and cardiovascular (HR 1.66; 95% CI 1.18–2.33; *P* = 0.003) mortality and compared to non-frailty T2DM. The positive association of frailty index and all-cause mortality was only in participants without CHF. The positive association of frailty index and cardiovascular mortality was only in non-anti-diabetic drug users.

**Conclusions:**

Frailty index in T2DM was positively associated with CHF in linear fashions. The Frailty index was positively correlated with all-cause and cardiovascular death in patients with T2DM. Frailty T2DM was positively associated with CHF, all-cause mortality, and cardiovascular mortality compared to non-frailty T2DM. Promoting frailty measurement and management in T2DM may be beneficial to reduce the burden of CHF and mortality.

**Supplementary Information:**

The online version contains supplementary material available at 10.1186/s13098-023-01165-z.

## Introduction

Type 2 diabetes (DM) is a temporary metabolic disease characterized by insulin resistance and β-cell dysfunction [1] that affects multiple organ systems, including the cardiovascular [2]. According to previous studies, heart failure may be the first cardiovascular manifestation of many T2DM patients, even without other cardiovascular diseases [3]. Besides, T2DM patients with heart failure have a worse prognosis [4]. The prevalence of HF in individuals with T2DM has up to 22% and will continue to rise [3]. This increase is mainly due to the increasing global prevalence of T2DM and an aging population [5, 6], making this co-morbid state more worrisome. Therefore, it is urgent to identify high-risk groups of heart failure in diabetic people for secondary prevention.

Frailty has attracted much attention in the field of diabetes in recent years. Firstly, the burden of frailty in T2DM patients is high due to their accelerated aging process [7, 8]. Secondly, frailty increases the adverse outcome of T2DM [9–11]. Recent studies have shown that despite optimal managing five cardiovascular risk factors, the risk of T2DM with heart failure was still high [12]. Understanding the independent effect of frailty on HF in patients with T2DM could help identify high-risk patients early and reduce mortality in the context of novel therapies.

Although the current definition of frailty is still inconsistent, Rockwood et al. [13] developed the FI model, which includes chronic diseases, psychosocial factors, cognitive deficits, and other signs and symptoms of old age, is the most commonly used model to assess frailty. The ratio of the accumulated acquired deficits to all potential deficits in the model calculates the frailty index [13]. The higher the deficit count, the frailer the person is. A standard FI-created procedure has shown repeatable properties and helped understand frailty-related health characteristics in older adults [14]. Another study demonstrated that FI was a prognostic survivorship factor in younger individuals and was confirmed to be validated by examining its distribution and associations with age and sex [15].

A previous study has demonstrated the excess risk of HF associated with FI in a nationally representative population [16]. A recent study confirmed higher frailty burdens were independently associated with a higher risk of cardiovascular diseases in patients with prediabetes and DM [17]. Still, the association of frailty index with congestive heart failure, all-cause, and cardiovascular mortality in diabetic patients throughout the U.S. population has not been thoroughly studied. The study was designed to explore the association of frailty with heart failure and death in diabetic patients in a large, nationally representative population.

## Research design and methods

### Study population

The National Health and Nutrition Examination Survey (NHANES) is an ongoing, cross-sectional, and nationally representative health survey. The NHANES was designed to assess the nutritional and health status of non-institutional civilians of the U.S. older than two months, conducted by the National Center for Health Statistics (NCHS) of the U.S. Centers for Disease Control and Prevention (CDC). And sampling methods and data collection details had been provided on the NHANES website (http://www.cdc.gov/nchs/nhanes (Accessed on 2 February 2023)].

**Fig. 1:**
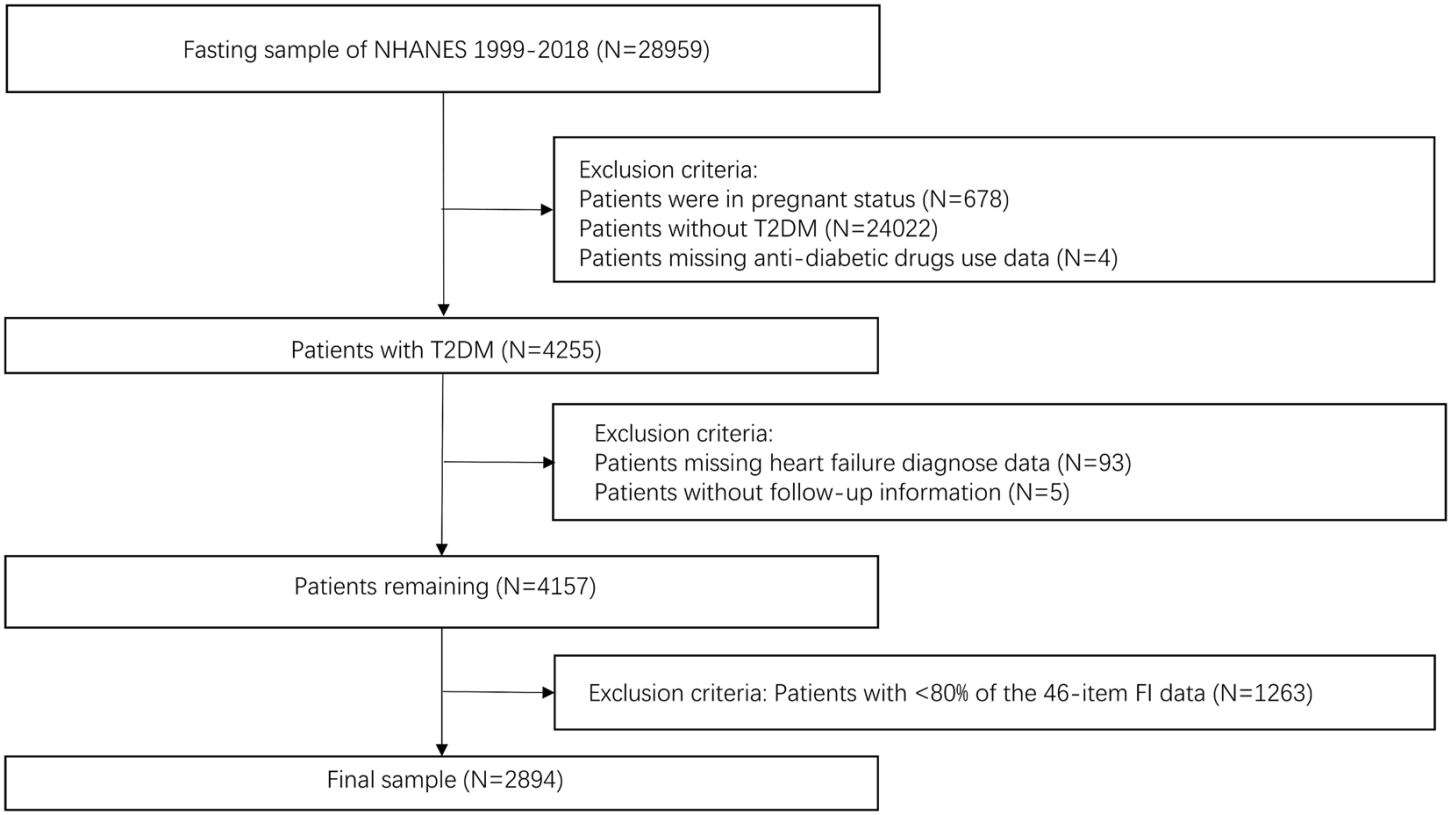
The flow chart of participants selection

The current study enrolled patients with T2DM in ten data cycles in NHANES from 1999 to 2018. As is shown in Fig. 1, participants without complete medical records or enough information (≥ 80% out of 46 items) for the calculation of FI were excluded. Finally, 2894 participants with National Death Index (NDI) mortality data to conduct the study.

### Definition of T2DM and CHF

Diabetes was defined as a self-reported diabetes diagnosis, use of diabetes medications or insulin, HbA1c ≥ 6.5%, fasting plasma glucose level ≥ 7.0 mmol/L, or random blood glucose (mmol/l) ≥ 11.1 mmol/L, 2-h OGTT blood glucose (mmol) ≥ 11.1 [18]. The definition of CHF was from the MCQ's positive answer: "Has a doctor or other health professional ever told you/sp that you/s/he had congestive heart failure?”.

### Frailty index

The 49-item FI used in previous studies covered different systems, including cognition, dependence, depression, comorbidities, hospital utilization and general health, anthropometry, and laboratory data [19], constructed according to standardized procedures published before [14]. Since our participants were strictly composed of T2DM, we excluded self-reported DM, glycohemoglobin, and self-reported CHF; finally, a 46-item frailty index was made. The final FI value ranges between 0 and 1; higher scores are presented with higher frailty. We also categorized our sample into two groups: non-frail and frail, and the cut-off FI value was 0.21 [20]. All items of FI are shown in (Additional file 1: Tables S1, S2).

### Covariates

Age, sex, race, education, smoking status, alcohol consumption, and medical history were self-reported. Race was categorized into Mexican American, non-Hispanic Black, non-Hispanic White, other Hispanic, and other Races; Smoking status was classified into three groups (no, former, and now). According to the self-reported average number of alcoholic drinks consumed daily, T2DM adults were classified as having no alcohol consumption, low to moderate alcohol consumption, or heavy alcohol consumption. A history of ASCVD was defined as coronary heart disease, angina, heart attack, and stroke. Hypertension was defined by those who had a self-reported history of hypertension, those who were taking anti-hypertensive medications, and those who were either having average systolic blood pressure (SBP) over 140 or/and average diastolic blood pressure (DBP) over 90 mmHg [21]. Hypertension can be diagnosed if one of these three criteria is met. Height and weight were collected at the mobile examination center (MEC), and BMI was calculated by the formula: body weight (kg)/ the square of height (m^2^). Obesity was defined as a BMI over 30 kg/m^2^. Laboratory methods for measurements of fasting total cholesterol (TC), high-density lipoprotein (HDL) cholesterol, fasting triglyceride (TG), fasting plasma glucose, albumin, and eGFR were reported in detail on the official NHANES website [http://www.cdc.gov/nchs/nhanes (Accessed on 2 February 2023)].

### Outcome

The outcome of this study was all-cause and cardiovascular death. Mortality data were ascertained by linkage to the National Death Index (NDI) through 31 December 2019. Cardiovascular mortality in this study was defined as death due to heart diseases (ICD codes I00–I09, I11, I13, I20–I51) and stroke (ICD codes I60–I69) according to the International Statistical Classification of Diseases, 10th Revision [22].

### Statistical analysis

We use complex sample weighted analysis of the fasting weights in the whole study according to NHANES analysis guidelines. T-test or Mann–Whitney *U* test (two groups of independent samples) were used to compare the continuous variables. Continuous variables are present as means (standard error). Categorical variables were expressed as numbers or percentages and compared by Pearson’s Chi-square test. Significant missing covariates were treated by missForest imputations when logistics and COX regression were ongoing to minimize the removal of samples. The logistic regression model was used to evaluate associations between FI and HF. The Cox regression models were constructed to assess the association of FI with all-cause and cardiovascular mortality. Two models were used in logistic and Cox regression analyses: model 1 was adjusted for age (continuous), gender, and race, and model 2 was adjusted for age (continuous), gender, race, education, smoking status, and alcohol consumption. Model 3 for logistic regression was adjusted for age (continuous), gender, race, education, smoking status, alcohol consumption, Obesity, systolic blood pressure, anti-diabetic drugs, HDL cholesterol, albumin, fasting plasma glucose, and eGFR. Model 3 for Cox regression was adjusted for age (continuous), gender, race, education, smoking status, alcohol consumption, Obesity, systolic blood pressure, anti-diabetic drugs, HDL cholesterol, albumin, fasting plasma glucose, eGFR, and CHF. Using a Cox proportional hazard model, the restricted cubic spline (RCS) further explored the relationship between FI and the outcome. The Kaplan–Meier method was used to analyze the survival probability data, and the log-rank test was used to compare the differences between each group. A fully adjusted multivariate regression model based on the interaction of FI with stratified covariates was used to conduct a subgroup analysis to assess whether the relationship between CHF and FI was affected by age (categories), sex, Obesity, and anti-diabetic drugs. When conducting a subgroup to determine the relationship between mortality and FI, we considered CHF in addition to the original four subgroups. Sensitivity analysis was conducted to determine the robustness by excluding participants whose follow-ups were less than one year or with an abnormal frailty index of Z score over ± 3. *P* < 0.05 was considered statistically significant. All statistical analyses were performed using R software (version 4.2).

## Results

### Baseline characteristics

The 2894 NHANES participants in T2DM with enough frailty index calculated information represented 19,493,702 non-institutionalized residents of the United States. Among the participants with T2DM [65.43 (0.30) years old; 49.23% males], the prevalence of CHF was 9.86%. Individuals with CHF were more likely to be older, had no alcohol consumption, and tended to have lower fasting total cholesterol, HDL cholesterol, albumin, and eGFR (Table 1). Patients with CHF were significantly associated with a higher percentage of Obesity, hypertension, ASCVD, anti-diabetic drug use, and frailty burden. At last, CHF patients had a higher frailty index (0.29 vs. 0.20, *P* < 0.0001), and weighted frailty prevalence was 40.40%.

**Table 1:** Baseline characteristics of participants with and without CHF

Characteristic	Total	CHF	Non-CHF	*P*-value
N (weighted)	2894(19,493,702)	290(1,922,401)	2604(17,571,301)	
Age, mean (SE)	65.43(0.30)	68.11(0.72)	65.14(0.32)	< 0.001
Male, % (SE)	1484(49.23)	156(52.81)	1328(48.84)	0.35
Race, % (SE)				0.16
Mexican American	509( 6.64)	38(6.24)	471(6.68)	
Non-Hispanic White	1223(68.05)	141(68.60)	1082(67.99)	
Non-Hispanic black	640(12.82)	76(16.52)	564(12.42)	
Other Hispanic	292( 5.65)	24(3.40)	268(5.90)	
Other Race	230( 6.83)	11(5.24)	219(7.00)	
Education, % (SE)				0.03
< High school	1079(25.62)	124(32.72)	955(24.85)	
High school graduate	693(27.50)	67(29.98)	626(27.24)	
College or higher	1118(46.84)	98(37.30)	1020(47.90)	
Smoking status, % (SE)				0.98
Never	1373(46.75)	125(47.48)	1248(46.68)	
Former	1093(38.51)	120(38.06)	973(38.57)	
Now	426(14.72)	45(14.46)	381(14.75)	
Alcohol consumption, % (SE)				< 0.001
None	1306(41.75)	157(59.60)	1149(43.65)	
Low to moderate	1074(41.83)	84(33.23)	990(46.67)	
Heavy	270(8.70)	25(7.17)	245(9.68)	
BMI, kg/m^2^, mean (SE)	31.90(0.18)	33.26(0.49)	31.76(0.18)	0.003
SBP, mmHg, mean (SE)	132.22(0.51)	132.52(1.47)	132.18(0.52)	0.82
DBP, mmHg, mean (SE)	68.14(0.36)	66.66(0.92)	68.29(0.37)	0.08
Fasting total cholesterol, mg/dL, mean (SE)	185.41(1.19)	177.37(3.96)	186.30(1.24)	0.03
HDL cholesterol, mg/dL, mean (SE)	49.73(0.42)	45.28(0.93)	50.22(0.43)	< 0.0001
Fasting Triglyceride, mg/dL, mean (SE)	164.86(3.69)	179.59(12.27)	163.24(3.90)	0.2
Albumin, g/dL, mean (SE)	4.13(0.01)	4.05(0.03)	4.14(0.01)	< 0.001
Fasting plasma glucose (mg/dL), mean (SE)	146.20(1.52)	144.06(4.08)	146.43(1.68)	0.6
eGFR, mean (SE)	77.10(0.58)	64.82(2.20)	78.45(0.59)	< 0.0001
Obesity, % (SE)	1500(54.17)	184(64.74)	1316(54.10)	0.01
Hypertension, % (SE)	2243(76.46)	257(88.11)	1986(75.18)	< 0.001
ASCVD, % (SE)	774(27.66)	209(71.09)	565(22.91)	< 0.0001
Anti-diabetic drugs, % (SE)	1704(57.59)	189(65.89)	1515(56.69)	0.02
Frailty index, mean (SE)	0.20(0.00)	0.29(0.01)	0.20(0.00)	< 0.0001
Frailty, % (SE)	1226(40.40)	227(71.61)	999(36.99)	< 0.0001

### The relationship of frailty index with CHF

**Fig. 2:**
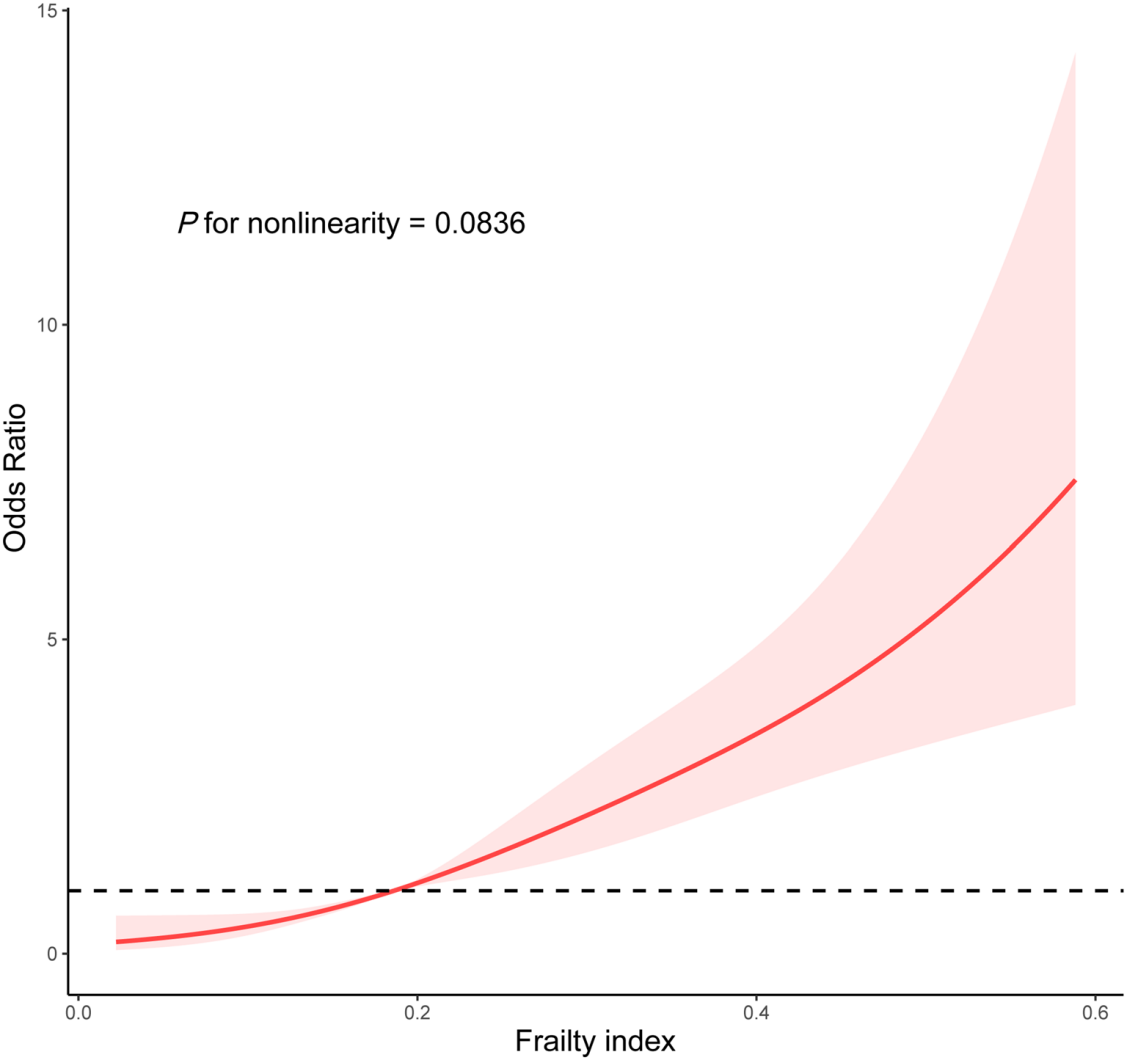
Restricted cubic spline plot of the association of FI with CHF. FI, Frailty index

**Fig. 3:**
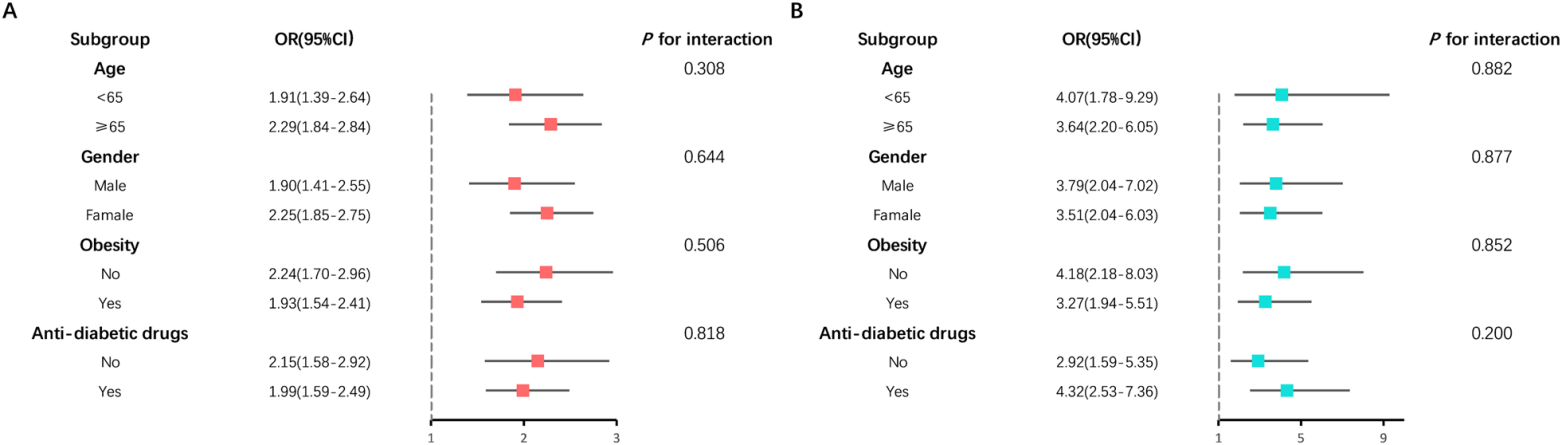
Forest plot of subgroup analysis for association of **A** FI and CHF, **B** frailty and CHF compared to non-frailty

We used weighted logistic regression to estimate the relationship of FI with CHF (Table 2). A crude analysis showed that an increase in FI was associated with a higher prevalence of CHF (OR = 2.10 1-SD, 95% CI 1.79–2.47, *P* < 0.0001). After adjusting for age (continuous), gender, race, education, smoking status, alcohol consumption, Obesity, systolic blood pressure, anti-diabetic drugs, HDL cholesterol, albumin, fasting plasma glucose, and eGFR, the association was still solid (OR = 2.02 1-SD, 95% CI 1.67–2.45, *P* < 0.0001). A weighted multivariate logistic regression was also conducted to examine the prevalence of CHF in T2DM participants with frailty. The presence of frailty T2DM (OR, 3.60; 95% CI 2.34–5.54; *P* < 0.0001) was associated with a significant increase in the prevalence of CHF compared to non-frailty T2DM in a fully adjusted model. As shown in Fig. 2, a restricted cubic spline was used to indicate the positive correlation of FI with CHF (*P* for nonlinearity = 0.0836). The overall population was stratified by age (categories), sex, Obesity, and anti-diabetic drugs, and subgroup analyses were conducted. We did not observe any significant interaction in both FI and frailty subgroup analysis (all *P* for interaction > 0.05, Fig. 3). The correlation was generally robust in sensitivity analysis when excluding participants with abnormal frailty index (Additional file 1: Table S3).

**Table 2:** Logistic regression analysis of the 1-SD increase in FI and frailty with CHF

	Crude model		Model 1		Model 2		Model 3	
	OR (95%CI)	*P*	OR (95%CI)	*P*	OR (95%CI)	*P*	OR (95%CI)	*P*
FI 1-SD increase	2.10(1.79,2.47)	< 0.0001	2.26(1.91,2.69)	< 0.0001	2.20(1.84,2.63)	< 0.0001	2.02(1.67,2.45)	< 0.0001
Non-frailty	Ref		Ref		Ref		Ref	
Frailty	4.30(2.80,6.59)	< 0.0001	4.63(3.03,7.09)	< 0.0001	4.42(2.89–6.77)	< 0.0001	3.60(2.34–5.54)	< 0.0001

Crude model: no covariates were adjusted

Model 1: age (continuous), gender, and race were adjusted

Model 2: age (continuous), gender, race, education, smoking status, and alcohol consumption were adjusted

Model 3: age (continuous), gender, race, education, smoking status, alcohol consumption, Obesity, systolic blood pressure, anti-diabetic drugs, HDL cholesterol, albumin, fasting plasma glucose, and eGFR were adjusted

### The relationship of the frailty index with all-cause and cardiovascular mortality

**Fig. 4:**
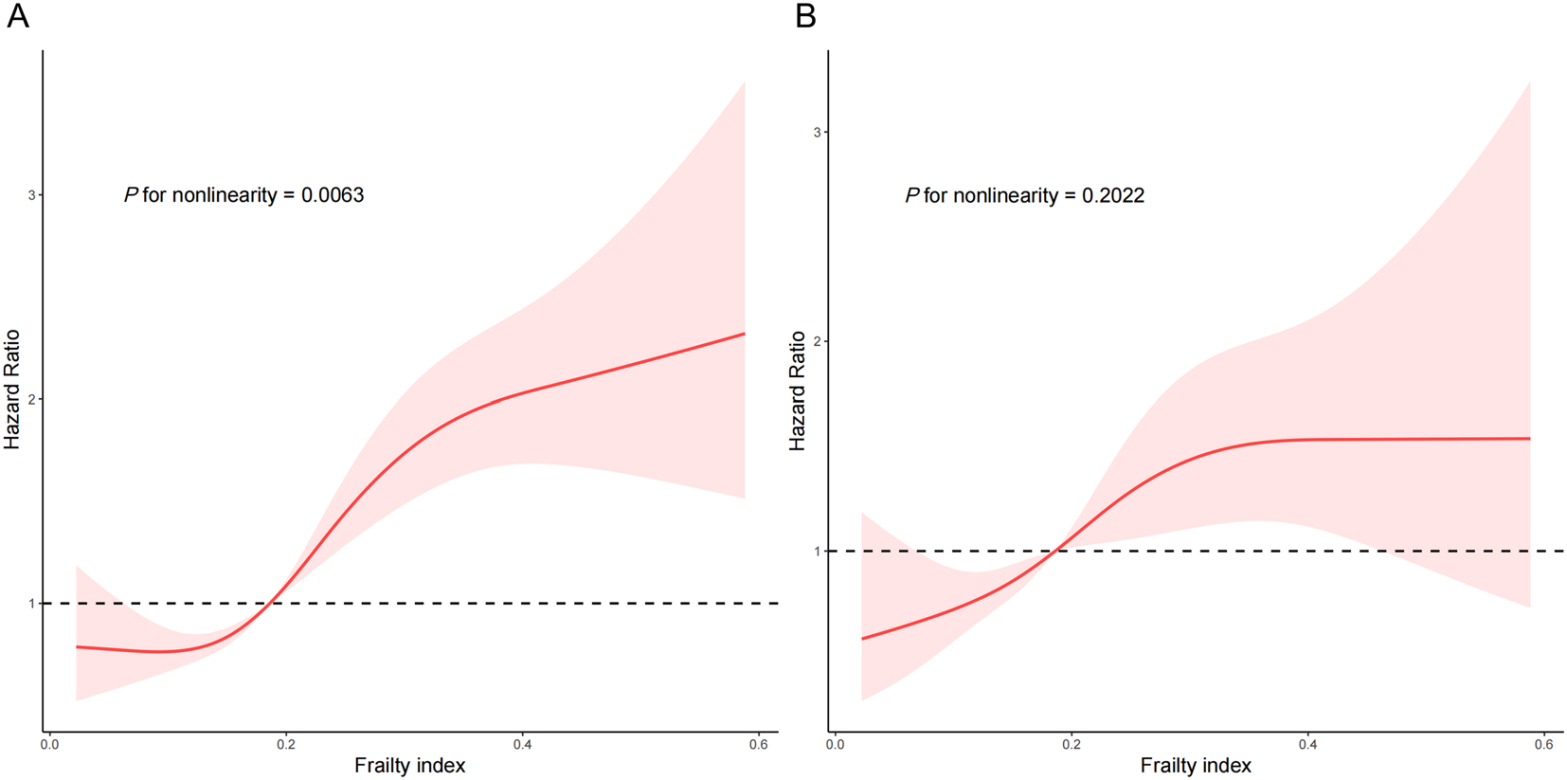
Kaplan–Meier curves of all-cause mortality and cardiovascular mortality based on FI. **A** Kaplan–Meier curves of all-cause mortality categorized by FI; **B** Kaplan–Meier curves of cardiovascular mortality categorized by FI

In this retrospective cohort, we observed an association of the frailty index with all-cause and cardiovascular mortality risk. Kaplan–Meier curves are shown in Fig. 4 to illustrate the survival probability according to non-frailty and frailty. Compared with the non-frailty group, individuals with frailty had the worst survival (36.34% at 20 years, log-rank test: P < 0.0001). The pattern was generally similar for cardiovascular mortality (log-rank test: P < 0.0001). Analysis of continuous FI showed that a 1-SD increase in FI was associated with a 44% higher risk of all-cause mortality without adjusting for confounders. The all-cause mortality risk persisted after adjusting for all confounders (HR = 1.41 1-SD, 95% CI  1.29–1.55, *P* < 0.0001). Similarly, a 1-SD increase in FI was associated with a 43% higher risk of cardiovascular mortality without adjusted and a 30% risk with fully adjusted for all confounders. Survival analysis was also conducted to examine the all-cause mortality and cardiovascular mortality between frailty T2DM and non-frailty T2DM. In a weighted multivariate COX proportional model adjusted for age (continuous), gender, race, education, smoking status, alcohol consumption, Obesity, systolic blood pressure, anti-diabetic drugs, HDL cholesterol, albumin, fasting plasma glucose, eGFR, and CHF, frailty T2DM increased all-cause (HR, 1.86; 95% CI 1.55–2.24; *P* < 0.0001) and cardiovascular (HR 1.66; 95% CI 1.18–2.33; *P* = 0.03) mortality and compared to non-frailty T2DM (Tables 3 and 4).

**Table 3:** Cox regression analysis of the 1-SD increase in FI and frailty with all-cause mortality

	Crude model		Model 1		Model 2		Model 3	
	HR (95%CI)	*P*	HR (95%CI)	*P*	HR (95%CI)	*P*	HR (95%CI)	*P*
FI 1-SD increase	1.44(1.32–1.56)	< 0.0001	1.57(1.44–1.70)	< 0.0001	1.50(1.38–1.64)	< 0.0001	1.41(1.29–1.55)	< 0.0001
Non-frailty	Ref		Ref		Ref		Ref	
Frailty	2.04(1.73–2.41)	< 0.0001	2.20(1.85–2.61)	< 0.0001	2.08(1.76–2.45)	< 0.0001	1.86(1.55–2.24)	< 0.0001

Crude model: no covariates were adjusted

Model 1: age (continuous), gender, and race were adjusted

Model 2: age (continuous), gender, race, education, smoking status, and alcohol consumption were adjusted

Model 3: age (continuous), gender, race, education, smoking status, alcohol consumption, Obesity, systolic blood pressure, anti-diabetic drugs, HDL cholesterol, albumin, fasting plasma glucose, eGFR, and CHF were adjusted

**Table 4:** Cox regression analysis of the 1-SD increase in FI and frailty with cardiovascular mortality

	Crude model		Model 1		Model 2		Model 3	
	HR (95%CI)	*P*	HR (95%CI)	*P*	HR (95%CI)	*P*	HR (95%CI)	*P*
FI 1-SD increase	1.40(1.23–1.58)	< 0.0001	1.52(1.33–1.73)	< 0.0001	1.46(1.28–1.67)	< 0.0001	1.30(1.10–1.54)	0.002
Non-frailty	Ref		Ref		Ref		Ref	
Frailty	1.95(1.46–2.60)	< 0.0001	2.09(1.57–2.78)	< 0.0001	2.00(1.51–2.65)	< 0.0001	1.66(1.18–2.33)	0.003

Crude model: no covariates were adjusted

Model 1: age (continuous), gender, and race were adjusted

Model 2: age (continuous), gender, race, education, smoking status, and alcohol consumption were adjusted

Model 3: age (continuous), gender, race, education, smoking status, alcohol consumption, Obesity, systolic blood pressure, anti-diabetic drugs, HDL cholesterol, albumin, fasting plasma glucose, eGFR, and CHF were adjusted

**Fig. 5:**
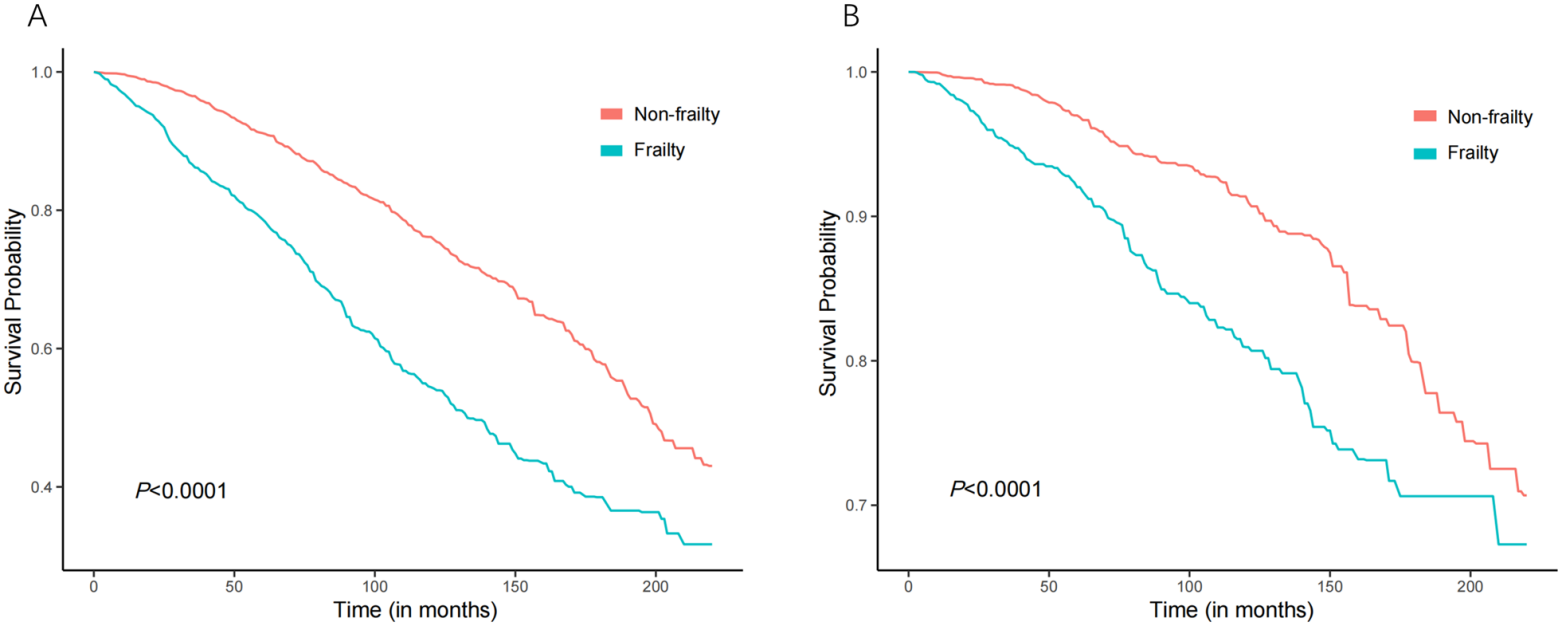
Restricted cubic spline plots of the association of FI with **A** all-cause mortality and **B** cardiovascular mortality. The results were adjusted for all covariates. FI, Frailty index

As shown in Fig. 5, restricted cubic splines were used to indicate the positive correlation of FI with all-cause and cardiovascular mortality. The relationship between FI and all-cause mortality was non-linear (*P* for nonlinearity = 0.0063). However, a non-linear relationship was not observed between FI and cardiovascular mortality (*P* for nonlinearity = 0.2022).

**Fig. 6:**
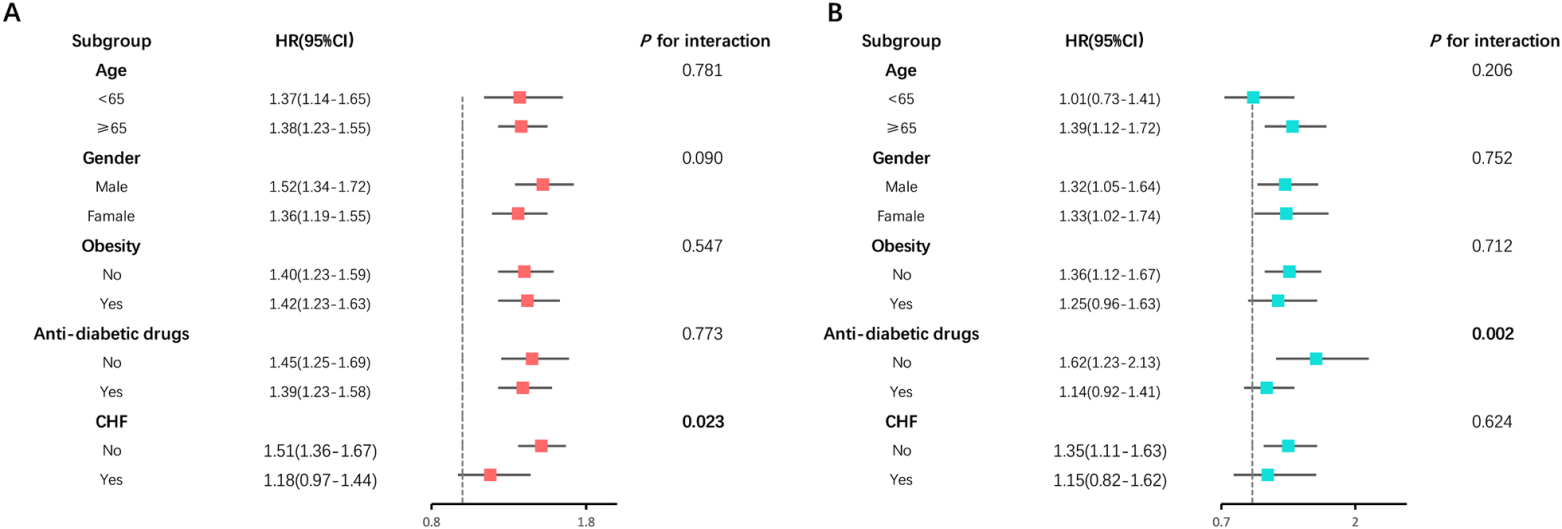
Forest plot of subgroup analysis of frailty index for **A** all-cause and **B** cardiovascular mortality

**Fig. 7:**
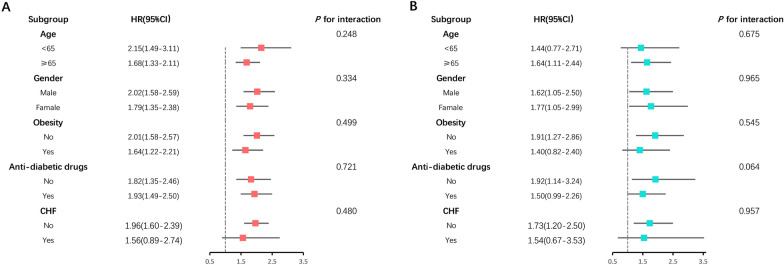
Forest plot of subgroup analysis for risk of **A** all-cause and **B** cardiovascular mortality in frailty compared to non-frailty

The overall population was stratified by age (categories), sex, Obesity, anti-diabetic drugs, and CHF, and subgroup analyses were conducted (Figs. 6 and 7). When the frailty index was treated as continuous, we did not observe any significant interaction in the all-cause mortality subgroup analysis except for the subgroups of CHF (*P* for interaction = 0.023). A significant interaction was identified between frailty index and anti-diabetic drug use for the cardiovascular mortality subgroup analysis. The inverse association between frailty index and cardiovascular mortality appeared only in non-anti-diabetic drug users (HR = 1.62 1-SD; 95% CI 1.23–2.13, *P* for interaction = 0.002). When the frailty index was categorized into non-frailty and frailty groups, we did not observe any significant interaction in the all-cause and cardiovascular mortality subgroup analysis (all *P* for interaction > 0.05).

The correlations were generally robust in sensitivity analysis when excluded participants whose follow-ups were less than one year or with abnormal frailty index (Additional file 1: Tables S2–S6).

## Discussion

This study explored the association of frailty index with heart failure, all-cause, and cardiovascular mortality in a representative sample of T2DM in the United States. Data collected from ten cycles of NHANES from 1999 to 2018 showed an association between frailty index and heart failure in patients with T2DM. The presence of frailty T2DM was associated with a significant increase in the prevalence of CHF compared to non-frailty T2DM. During the median follow-up of 6.75 years, increased FI was positively associated with all-cause and cardiovascular mortality. Along with the 1-SD increase in FI, the risk of all-cause mortality showed a 41% increase. In the meantime, the increase in cardiovascular mortality risk was 30%. Compared to non-frailty. Frailty T2DM was associated with a significant increase in the risk of all-cause and cardiovascular death compared to non-frailty T2DM. The positive association of frailty index and all-cause mortality was only in participants with CHF, and cardiovascular mortality was only in non-anti-diabetic users when FI was continuous.

The Fried phenotype and the Rockwood index are classic models for assessing frailty status [13, 23]. The Fried phenotype assesses the impairment of physiological reserves in 5 areas of body function: unintentional weight loss, loss of endurance, frailty, slowness of movement, and low levels of physical activity. Frailty refers to people with more than two of the above factors [23]. Rockwood FI assesses frailty as an accumulation of health deficits in multiple domains. It can be evaluated using clinical and health status data on signs and symptoms, comorbidities, laboratory data, activities of daily living, and patient-reported symptoms. Its construction model is flexible and evolving [13]. Many studies have assessed their similarities and differences, but there is still no complete agreement [20, 24–27]. Some studies prove that the vulnerability index can define the risk of adverse outcomes more precisely than phenotypes [28–31]. Several extensive studies and clinical trials in diabetic populations have also assessed frailty using the cumulative frailty index of health defects^32^–^34^. Thus, we ultimately chose to evaluate frailty in diabetes patients by cumulatively computing a frailty index for multiple domain health deficits.

Our study indicated that FI is associated with an increased prevalence of heart failure in T2DM. It is consistent with a recent post hoc analysis of prospective trials in which higher baseline frailty was significantly associated with a higher risk of HF in adults with T2DM [17]. The retrospective study by Chen et al. showed that frailty was associated with an increased prevalence of CHF, and subgroup analysis showed elevated FI in HF patients with T2DM. Still, no further studies were conducted [16].

Our findings confirm the positive association of frailty with all-cause mortality and cardiovascular death in patients with diabetes. Consistent with previous studies, one study showed a positive association of frailty with all-cause mortality in middle-aged and older diabetic patients [35, 36]. Also, in two prospective cohorts, frailty has been confirmed to be associated with the progression of elevated risks of all-cause mortality in individuals with prediabetes and diabetes [37]. Our study also found that frailty was associated with cardiovascular mortality in patients with T2DM in this large, nationally representative population.

The connection between the severity of frailty and mortality risk in T2DM was inconclusive. A non-linear relationship between FI and all-cause mortality in patients with T2DM was observed in the RCS, not cardiovascular mortality. According to our study, the median of FI for all-cause and cardiovascular mortality was 0.186. Our study provides a reference for the median FI of T2DM in the community. Still, it should be interpreted cautiously because the proportion of T2DM patients excluded due to insufficient FI data is not low, and it is likely to have selection bias. Our study determined the FI cut-off value of 0.21 in the diabetic population was useful and provided a reliable basis for identifying and managing frail patients with T2DM. Moreover, we also found that KM survival curves were distinguished significantly by grouping with a cut-off of 0.21. The stability of this cut-off value was further verified.

In the subgroup analysis for all-cause mortality with FI, the association between frailty index and all-cause mortality appeared only in participants without a CHF history. This suggests that the risk of all-cause death from diabetes with frailty and other complications or comorbidities may not necessarily be lower than that of heart failure. And the severity of heart failure may mask the effect of frailty on all-cause death. It also suggests that it may be beneficial to study the potential benefits of heart failure treatment for debilitating type 2 diabetes. In the subgroup analysis for cardiovascular mortality with FI, the association between frailty index and cardiovascular mortality appeared only in non-anti-diabetic drug users. This suggests that using hypoglycemic drugs may mitigate the link between frailty and cardiovascular death in patients with type 2 diabetes through blood sugar control, prevention of cardiac complications, or other possible disease-regulating effects. Moreover, specific new hypoglycemic drugs have also shown the effect of resisting weak cardiovascular damage and have good safety [38]. More research is needed to confirm this exciting association.

The mechanism of frailty associated with heart failure and mortality in T2DM remains unclear. The pathophysiological mechanisms of frailty and heart failure are intertwined, including upregulation of pro-inflammatory states, metabolic impairment, and insulin resistance [39]. Increased risk of incident heart failure and death is associated with insulin resistance in people with newly diagnosed type 2 diabetes [40]. Frailty was considered a significant factor in increasing the risk for hypoglycemia [41]. A recent randomized controlled trial confirmed severe hypoglycemia was independently associated with a greater risk of incident HF [42]. Another recent study indicated that severe hypoglycemia was associated with increased risks of hospitalization for HF among adults with diabetes regardless of coexistent CVDs [43]. These findings suggest that hypoglycemia may be an intermediary in T2DM between frailty and heart failure. More interestingly, a previous study showed that incremental frailty was associated with increased hyperglycemia rather than hypoglycemia in older adults with T2DM who are on insulin therapy. HR was increased with severe hyperglycemia [44]. Frailty was also associated with cognitive impairment. Cognitive impairment was a decisive prognostic factor identifying people with diabetes at high risk of mortality [45]. Both Frailty and hypoglycemia increased functional decline, leading to a gradient effect on mortality in individuals with diabetes [46]. Moreover, frailty may also reduce the protective effect of certain anti-diabetic drugs, such as metformin, on adverse outcomes [47]. Therefore, further mechanism exploration is urgently needed.

The study provides new insights into CHF and death associated with frailty in people with T2DM. First, the study showed a need for frailty assessment in patients with T2DM in America. It is well-known that frailty is a geriatric syndrome. The previous clinical guidelines and consensus report advocate that frailty assessment is an essential component of diabetes management for older patients [48, 49]. However, our findings suggested the need for frailty assessment was not only for older patients but also for individuals under 65 years to prevent CHF and death. Second, for patients with T2DM without heart failure, future explorations should also actively identify or manage flimsy to reduce the risk of all-cause death. Third, to study the potential protective mechanism of different hypoglycemic drugs on the effects of T2DM weakness on cardiovascular death and prescribing appropriate personalized hypoglycemic prescriptions may be a vital management link in the study of T2DM-frailty. Furthermore, due to the predictive value and the dynamic nature of frailty [9, 10, 17, 50, 51], multifactorial interventions will be needed to reverse frailty or delay the further progression of diabetes, including optimal nutrition with protein intake, combining aerobic, weight-bearing, and resistance training [52]. Relevant clinical trials are also needed to explore appropriate treatment strategies to guide treatment decisions.

Our study also has some limitations. First, we could not infer causality attributable to the observational study design. Our findings need further prospective studies with large samples to confirm. Second, we only considered FI at baseline and did not have data on dynamic changes in FI, which may lead to bias. In addition, although we considered as many covariates as possible, some confounding factors still have not been adjusted.

## Conclusion

In this nationally representative sample of US adults, the frailty index in T2DM was positively associated with the presence of CHF in non-linear fashions. The Frailty index was positively correlated with all-cause and cardiovascular death in patients with T2DM. Frailty T2DM was positively associated with CHF, all-cause mortality, and cardiovascular mortality compared to non-frailty T2DM. Moreover, the strength of the association between FI and mortality differed within the study population. Promoting frailty measurement and management in T2DM may be beneficial to reduce the burden of CHF and mortality.

### Supplementary Information

**Additional file 1: Table S1.** Variables in the 46-item frailty index and their respective scorings. **Table S2.** Logistic regression analysis of the 1-SD FI and frailty after excluding participants with abnormal FI data. (N = 2873). **Table S3.** Risk of all-cause mortality among diabetes patients according to 1-SD FI and frailty after excluding participants who died within 1 years of follow-up (N = 2834). **Table S4.** Risk of cardiovascular mortality among diabetes patients according to 1-SD FI and frailty after excluding participants who died within 1 years of follow-up (N = 2834). **Table S5.** Risk of all-cause mortality among diabetes patients according to 1-SD FI and frailty after excluding participants with abnormal FI data (N = 2873). **Table S6.** Risk of cardiovascular mortality among diabetes patients according to 1-SD FI and frailty after excluding participants with abnormal FI data (N = 2873).

## Data Availability

The original contributions presented in the study are included in the article/Additional files. Further inquiries can be directed to the corresponding authors.
