# Supplementary material for: Association of frailty index with congestive heart failure, all-cause and cardiovascular mortality among individuals with type 2 diabetes: a study from National Health and Nutrition Examination Surveys (NHANES), 1999–2018
Source: Diabetol Metab Syndr. 2023 Oct 24;15:210. doi: 10.1186/s13098-023-01165-z (PMC10594933; doi:10.1186/s13098-023-01165-z)
Supplement: Supplementary file 1 — Additional file 1: Table S1. Variables in the 46-item frailty index and their respective scorings. Table S2. Logistic regression analysis of the 1-SD FI and frailty after excluding participants with abnormal FI data. (N = 2873). Table S3. Risk of all-cause mortality among diabetes patients according to 1-SD FI and frailty after excluding participants who died within 1 years of follow-up (N = 2834). Table S4. Risk of cardiovascular mortality among diabetes patients according to 1-SD FI and frailty after excluding participants who died within 1 years of follow-up (N = 2834). Table S5. Risk of all-cause mortality among diabetes patients according to 1-SD FI and frailty after excluding participants with abnormal FI data (N = 2873). Table S6. Risk of cardiovascular mortality among diabetes patients according to 1-SD FI and frailty after excluding participants with abnormal FI data (N = 2873). [file 13098_2023_1165_MOESM1_ESM.docx]

Table S1. Variables in the 46-item frailty index and their respective scorings

| **Cognition** |  |
| --- | --- |
| 1.Experience confusion/memory problems | Yes=1, No=0 |
| **Dependence** |  |
| 2.Managing money | Difficulty=1, No Difficulty=0 |
| 3.Stooping, crouching, kneeling | Difficulty=1, No Difficulty=0 |
| 4.Lifting or carrying | Difficulty=1, No Difficulty=0 |
| 5.House chore | Difficulty=1, No Difficulty=0 |
| 6.Preparing meals | Difficulty=1, No Difficulty=0 |
| 7.Standing up from armless chair | Difficulty=1, No Difficulty=0 |
| 8.Getting in and out of bed difficulty | Difficulty=1, No Difficulty=0 |
| 9.Using fork, knife, drinking from cup | Difficulty=1, No Difficulty=0 |
| 10.Dressing yourself | Difficulty=1, No Difficulty=0 |
| 11.Standing for long periods difficulty | Difficulty=1, No Difficulty=0 |
| 12.Grasp/holding small objects | Difficulty=1, No Difficulty=0 |
| 13.Attending social event | Difficulty=1, No Difficulty=0 |
| 14.Push or pull large objects | Difficulty=1, No Difficulty=0 |
| 15.walking for a quarter mile difficulty | Difficulty=1, No Difficulty=0 |
| 16.walking up 10 steps difficulty | Difficulty=1, No Difficulty=0 |
| 17.Leisure activity at home difficulty | Difficulty=1, No Difficulty=0 |
| **Depressive Symptoms** |  |
| 18.Have little interest in doing things | Nearly every day=1, More than half the days=0.66, Several days=0.33, Not at all=0 |
| 19.Feeling down, depressed, or hopeless | Nearly every day=1, More than half the days=0.66, Several days=0.33, Not at all=0 |
| 20.Trouble sleeping or sleeping too much | Nearly every day=1, More than half the days=0.66, Several days=0.33, Not at all=0 |
| 21.Feeling tired or having little energy | Nearly every day=1, More than half the days=0.66, Several days=0.33, Not at all=0 |
| 22.Poor appetite or overeating | Nearly every day=1, More than half the days=0.66, Several days=0.33, Not at all=0 |
| 23.Feeling bad about yourself | Nearly every day=1, More than half the days=0.66, Several days=0.33, Not at all=0 |
| 24.Trouble concentrating on things | Nearly every day=1, More than half the days=0.66, Several days=0.33, Not at all=0 |
| **Comorbidities** |  |
| 25.Arthritis | Yes=1, Suspect=0.5, No=0 |
| 26.Thyroid problems | Yes=1, Suspect=0.5, No=0 |
| 27.Chronic bronchitis | Yes=1, Suspect=0.5, No=0 |
| 28.Cancer | Yes=1, Suspect=0.5, No=0 |
| 29.Coronary heart disease | Yes=1, Suspect=0.5, No=0 |
| 30.Angina | Yes=1, Suspect=0.5, No=0 |
| 31.Heart attack | Yes=1, Suspect=0.5, No=0 |
| 32.Stroke | Yes=1, Suspect=0.5, No=0 |
| 33.High blood pressure | Yes=1, Suspect=0.5, No=0 |
| 34.Weak/failing kidneys | Yes=1, Suspect=0.5, No=0 |
| 35.Urinary leakage | Yes=1, Suspect=0.5, No=0 |
| **Hospital Utilization and Access to Care** |  |
| 36.Self-rated health | Fair, poor=1, Excellent, Very good, good=0 |
| 37.Health now compared 1 year ago | Worse=1, About the same, Better=0 |
| 38.Overnight hospital patient in past year | Yes=1, No=0 |
| 39.Frequency of healthcare using during past year | None=0, 1-5=0.5, 5 and more than 5=1 |
| 40.Number of prescribed medications | None=0, 1-4=0.5, More than 5=1 |
| **Physical Performance and Anthropometry** |  |
| 41.Body mass index | <18.5, ≥30=1  25-<30=0.5  18.5-25=0 |
| **Laboratory Values** |  |
| 42.Red blood cell count (million cells/μL) | M: 4.7-6.1=0, Other=1; F: 4.2-5.4=0, Other=1 |
| 43.Hemoglobin (g/dL) | M:13.5-18=0, Other=1; F: 12-16=0, Other=1 |
| 44.Red cell distribution width (%) | 11.6-14.6=0, Other=1 |
| 45.Lymphocyte percent(%) | 20-40=0, Other=1 |
| 46.Segmented neutrophils percent(%) | 40-80=0, Other=1 |

Table S2. Logistic regression analysis of the 1-SD FI and frailty after excluding participants with abnormal FI data. (N=2873).

|  | Crude Model | |  | Model 1 | |  | Model 2 | |  | Model 3 | |
| --- | --- | --- | --- | --- | --- | --- | --- | --- | --- | --- | --- |
|  | OR (95%CI) | *P* |  | OR (95%CI) | *P* |  | OR (95%CI) | *P* |  | OR (95%CI) | *P* |
| FI  1-SD increase | 2.11(1.78-2.52) | <0.0001 |  | 2.26(1.89-2.71) | <0.0001 |  | 2.21(1.83-2.67) | <0.0001 |  | 2.02(1.65-2.49) | <0.0001 |
| Non-frailty | Ref |  |  | Ref |  |  | Ref |  |  | Ref |  |
| Frailty | 4.20(2.73-6.46) | <0.0001 |  | 4.52(2.95-6.94) | <0.0001 |  | 4.34(2.82-6.68) | <0.0001 |  | 3.56(2.30-5.50) | <0.0001 |

Crude model: no covariates were adjusted

Model 1: age (continuous), gender, and race were adjusted

Model 2: age (continuous), gender, race, education, smoking status, and alcohol consumption were adjusted

Model 3: age (continuous), gender, race, education, smoking status, alcohol consumption, Obesity, systolic blood pressure, anti-diabetic drugs, HDL cholesterol, albumin, fasting plasma glucose, and eGFR were adjusted

Table S3. Risk of all-cause mortality among diabetes patients according to 1-SD FI and frailty after excluding participants who died within 1 years of follow-up (N=2834).

|  | Crude Model | |  | Model 1 | |  | Model 2 | |  | Model 3 | |
| --- | --- | --- | --- | --- | --- | --- | --- | --- | --- | --- | --- |
|  | HR (95%CI) | *P* |  | HR (95%CI) | *P* |  | HR (95%CI) | *P* |  | HR (95%CI) | *P* |
| FI  1-SD increase | 1.40(1.27-1.53) | <0.0001 |  | 1.52(1.39-1.65) | <0.0001 |  | 1.47(1.34-1.60) | <0.0001 |  | 1.38(1.26-1.51) | <0.0001 |
| Non-frailty | Ref |  |  | Ref |  |  | Ref |  |  | Ref |  |
| Frailty | 1.92(1.61-2.28) | <0.0001 |  | 2.05(1.72-2.45) | <0.0001 |  | 1.96(1.65-2.32) | <0.0001 |  | 1.76(1.46-2.12) | <0.0001 |

Crude model: no covariates were adjusted

Model 1: age (continuous), gender, and race were adjusted

Model 2: age (continuous), gender, race, education, smoking status, and alcohol consumption were adjusted

Model 3: age (continuous), gender, race, education, smoking status, alcohol consumption, Obesity, systolic blood pressure, anti-diabetic drugs, HDL cholesterol, albumin, fasting plasma glucose, eGFR, and CHF were adjusted

Table S4. Risk of cardiovascular mortality among diabetes patients according to 1-SD FI and frailty after excluding participants who died within 1 years of follow-up (N=2834).

|  | Crude Model | |  | Model 1 | |  | Model 2 | |  | Model 3 | |
| --- | --- | --- | --- | --- | --- | --- | --- | --- | --- | --- | --- |
|  | HR (95%CI) | *P* |  | HR (95%CI) | *P* |  | HR (95%CI) | *P* |  | HR (95%CI) | *P* |
| FI  1-SD increase | 1.35(1.19-1.54) | <0.0001 |  | 1.46(1.28-1.65) | <0.0001 |  | 1.42(1.25-1.62) | <0.0001 |  | 1.27(1.07-1.51) | 0.01 |
| Non-frailty | Ref |  |  | Ref |  |  | Ref |  |  | Ref |  |
| Frailty | 1.86(1.38-2.52) | <0.001 |  | 1.97(1.47-2.64) | <0.0001 |  | 1.91(1.43-2.55) | <0.0001 |  | 1.62(1.14-2.31) | 0.01 |

Crude model: no covariates were adjusted

Model 1: age (continuous), gender, and race were adjusted

Model 2: age (continuous), gender, race, education, smoking status, and alcohol consumption were adjusted

Model 3: age (continuous), gender, race, education, smoking status, alcohol consumption, Obesity, systolic blood pressure, anti-diabetic drugs, HDL cholesterol, albumin, fasting plasma glucose, eGFR, and CHF were adjusted

Table S5. Risk of all-cause mortality among diabetes patients according to 1-SD FI and frailty after excluding participants with abnormal FI data (N=2873).

|  | Crude Model | |  | Model 1 | |  | Model 2 | |  | Model 3 | |
| --- | --- | --- | --- | --- | --- | --- | --- | --- | --- | --- | --- |
|  | HR (95%CI) | *P* |  | HR (95%CI) | *P* |  | HR (95%CI) | *P* |  | HR (95%CI) | *P* |
| FI  1-SD increase | 1.48(1.37-1.60) | <0.0001 |  | 1.57(1.45-1.71) | <0.0001 |  | 1.51(1.40-1.64) | <0.0001 |  | 1.42(1.30-1.55) | <0.0001 |
| Non-frailty | Ref |  |  | Ref |  |  | Ref |  |  | Ref |  |
| Frailty | 2.06(1.74-2.43) | <0.0001 |  | 2.19(1.84-2.60) | <0.0001 |  | 2.08(1.76-2.45) | <0.0001 |  | 1.87(1.55-2.26) | <0.0001 |

Crude model: no covariates were adjusted

Model 1: age (continuous), gender, and race were adjusted

Model 2: age (continuous), gender, race, education, smoking status, and alcohol consumption were adjusted

Model 3: age (continuous), gender, race, education, smoking status, alcohol consumption, Obesity, systolic blood pressure, anti-diabetic drugs, HDL cholesterol, albumin, fasting plasma glucose, eGFR, and CHF were adjusted

Table S6. Risk of cardiovascular mortality among diabetes patients according to 1-SD FI and frailty after excluding participants with abnormal FI data (N=2873).

|  | Crude Model | |  | Model 1 | |  | Model 2 | |  | Model 3 | |
| --- | --- | --- | --- | --- | --- | --- | --- | --- | --- | --- | --- |
|  | HR (95%CI) | *P* |  | HR (95%CI) | *P* |  | HR (95%CI) | *P* |  | HR (95%CI) | *P* |
| FI  1-SD increase | 1.45(1.27-1.66) | <0.0001 |  | 1.54(1.34-1.77) | <0.0001 |  | 1.49(1.29-1.71) | <0.0001 |  | 1.32(1.10-1.57) | 0.002 |
| Non-frailty | Ref |  |  | Ref |  |  | Ref |  |  | Ref |  |
| Frailty | 1.98(1.48-2.65) | <0.0001 |  | 2.10(1.57-2.80) | <0.0001 |  | 2.01(1.51-2.67) | <0.0001 |  | 1.67(1.19,2.35) | 0.003 |

Crude model: no covariates were adjusted

Model 1: age (continuous), gender, and race were adjusted

Model 2: age (continuous), gender, race, education, smoking status, and alcohol consumption were adjusted

Model 3: age (continuous), gender, race, education, smoking status, alcohol consumption, Obesity, systolic blood pressure, anti-diabetic drugs, HDL cholesterol, albumin, fasting plasma glucose, eGFR, and CHF were adjusted
